# Spatio-Temporal Collaborative Perception-Enabled Fault Feature Graph Construction and Topology Mining for Variable Operating Conditions Diagnosis

**DOI:** 10.3390/s25154664

**Published:** 2025-07-28

**Authors:** Jiaxin Zhao, Xing Wu, Chang Liu, Feifei He

**Affiliations:** 1 Key Laboratory of Advanced Equipment Intelligent Manufacturing Technology of Yunnan Province, Kunming University of Science & Technology, Kunming 650500, China; z1847718436@163.com (J.Z.); xwu@kust.edu.cn (X.W.); heff0523@163.com (F.H.); 2 Faculty of Mechanical & Electrical Engineering, Kunming University of Science &Technology, Kunming 650500, China; 3 West Yunnan University of Applied Sciences, Dali 671000, China

**Keywords:** feature topology, spatio-temporal collaborative perception fusion, graph neural network, fault feature mining

## Abstract

Industrial equipment fault diagnosis faces dual challenges: significant data distribution discrepancies caused by diverse operating conditions impair generalization capabilities, while underutilized spatio-temporal information from multi-source data hinders feature extraction. To address this, we propose a spatio-temporal collaborative perception-driven feature graph construction and topology mining methodology for variable-condition diagnosis. First, leveraging the operational condition invariance and cross-condition consistency of fault features, we construct fault feature graphs using single-source data and similarity clustering, validating topological similarity and representational consistency under varying conditions. Second, we reveal spatio-temporal correlations within multi-source feature topologies. By embedding multi-source spatio-temporal information into fault feature graphs via spatio-temporal collaborative perception, we establish high-dimensional spatio-temporal feature topology graphs based on spectral similarity, extending generalized feature representations into the spatio-temporal domain. Finally, we develop a graph residual convolutional network to mine topological information from multi-source spatio-temporal features under complex operating conditions. Experiments on variable/multi-condition datasets demonstrate the following: feature graphs seamlessly integrate multi-source information with operational variations; the methodology precisely captures spatio-temporal delays induced by vibrational direction/path discrepancies; and the proposed model maintains both high diagnostic accuracy and strong generalization capacity under complex operating conditions, delivering a highly reliable framework for rotating machinery fault diagnosis.

## 1. Introduction

Rotating equipment serves as the cornerstone of industrial production and is widely utilized in sectors such as power generation, petrochemicals, and manufacturing. However, prolonged operation can lead to issues like wear and tear, lubrication failure, and imbalance, which may trigger malfunctions. In minor cases, these can result in shutdowns, production delays, and financial losses, while severe cases may even compromise safety. Therefore, investigating fault detection in rotating machinery is critical.

Amidst the swift advancement of automated and intelligent industry, traditional fault diagnosis methods relying on manual expertise are not only inefficient but also susceptible to subjective interference. These methods can no longer meet the stringent reliability requirements demanded by modern industry. Against this backdrop, fault diagnosis techniques that integrate advanced sensor technology, signal analysis, deep learning, and artificial intelligence have gained prominence. These techniques have become crucial tools for enhancing equipment reliability and maintenance efficiency. Early research efforts primarily concentrated on signal analysis approaches. As an example, Pan et al. [1] introduced an improved symplectic geometry mode decomposition method. This approach, incorporating the Ramanujan spectrum signal-to-noise ratio index, achieves multi-level decomposition and denoising of stationary signals, resulting in superior noise suppression and feature extraction capabilities. Wang et al. [2] introduced an enhanced version of the feature mode decomposition method. This approach adaptively determines critical parameters and optimizes the decomposition process, improving early fault detection precision in planetary gearboxes. However, conventional signal analysis and feature extraction methods often rely heavily on expert experience and involve intricate signal processing workflows. With the progress of deep learning methods, research focusing on application in fault diagnosis has grown significantly. For example, Wen et al. [3] converted 1D signals into time-frequency images via continuous wavelet transform (WT) and employed an improved dilated convolutional neural network (CNN) for single-condition fault diagnosis in gearboxes. Tang et al. [4] designed a CNN model optimized with the Nadam algorithm, deepening the network structure to enhance feature learning capability for simple-condition fault diagnosis in rolling bearings. Duan et al. [5] developed a hybrid approach combining CNN attention mechanisms with Transformer-based temporal attention, integrating local and global features while weighting their importance to improve diagnostic performance. Wang et al. [6] proposed a method combining the wavelet packet pooling mechanism and the biological interpretability of spiking neural networks, effectively tackling the challenge of feature extraction for bearings and cutting tools, and improving the robustness of fault diagnosis. Nevertheless, these approaches primarily focus on single-source data, limiting their ability to characterize equipment states under complex operating conditions.

To break through this bottleneck, multi-source data fusion methods have emerged, integrating data from diverse channels, sensors, and operating conditions to extract comprehensive equipment state representations. For instance, Ji et al. [7] treated vibration data from three different sensor positions as multi-channel inputs, employing a multi-scale dual–quadratic convolutional neural network (MDQCNN) to extract low, medium, and high frequency, followed by feature fusion using a cross-attention mechanism. Song et al. [8] encoded multi-channel data as grayscale images through continuous wavelet transform (CWT) and grayscale processing, then performed weighted fusion of these images using a channel attention mechanism. Qiao et al. [9] developed a time-distributed 3D convolutional layer (TD-Conv3D) that adaptively assigns weights to features extracted from different sensor signals through a weight vector generation approach. However, when confronted with complex equipment structures and dynamic operating conditions, these data fusion methods exhibit limitations in uncovering intrinsic correlations and deep dependencies among heterogeneous data sources. This remains a critical challenge in the field of equipment fault diagnosis.

In this context, graph neural networks (GNNs) have established themselves as a revolutionary methodology for fault diagnosis, owing to their distinctive capacity to process graph-structured data and capture complex node relationships. For instance, Liu et al. [10] introduced an information-theoretic graph causal learning graph neural network (IGCL-GNN), which transforms the classification problem into a multi-objective optimization task. By enhancing causal feature extraction, this approach addresses the limitation of conventional GNN models that rely on non-causal features, thereby overcoming their insufficient generalization capability. Li et al. [11] introduced a multi-view graph neural network (MvGNN) that employs two independent graph convolutional blocks to separately process time-domain and frequency-domain views, followed by feature fusion through a view attention module. This approach effectively addresses the limitation of existing methods in fully characterizing complex inter-sensor signal relationships. Yan et al. [12] developed a multi-path parallel graph convolutional residual network (MPGCN-ResNet) that utilizes multi-scale convolutional kernels to capture features across different receptive fields, while utilizing residual structures for deep feature mining. This architecture significantly enhances both the accuracy and robustness of fault diagnosis. Additionally, Chen et al. [13] designed an enhanced perception graph neural network (E-GCN) that incorporates dilated convolution blocks to significantly improve the model’s comprehension capability for graph-structured data. This innovative methodology successfully overcomes the critical challenge of limited generalization under complex noise environments and variable operating conditions. However, current GNN methods still face challenges in handling multi-source time-series data, including difficulties in modeling temporal dynamics and spatial dependencies, reliance on manually designed graph structures that may compromise computational efficiency and information integrity, and susceptibility to overfitting when processing large-scale graphs, which limits their practical application in complex industrial scenarios.

To address these challenges, this paper proposes a spatio-temporal collaborative perception-driven multi-source feature graph construction and topology mining method for variable operating condition diagnosis. Firstly, intrinsic feature relationships are autonomously explored through similarity clustering to construct a feature topology graph possessing condition invariance, achieving robust and consistent representation of fault features under complex operating conditions such as variable speeds and variable loads, thereby mitigating intra-class condition variations. Secondly, a spatio-temporal cooperative fusion technique driven by spectral similarity is proposed. By integrating spatio-temporal coupling information from multi-source features, a high-dimensional spatio-temporal feature topology graph is constructed, resolving spatial alignment issues through collaborative perception modeling across spatio-temporal scales. Finally, a graph residual convolutional network is designed to mine feature information via spatio-temporal topology adaptive learning mechanisms, achieving precise diagnosis of multi-type compound faults under complex operational conditions.

Compared with existing methods, the approach proposed in this paper breaks through the limitations of traditional single-dimensional feature extraction and demonstrates stronger feature generalization capabilities and fault identification abilities under challenges such as complex operating conditions and spatio-temporal vibrations.

The main contributions of this study are summarized as follows:

(1) The proposed single-source feature topology graph achieves consistent feature representation across operating conditions through dynamic neighborhood optimization, effectively eliminating feature drift caused by condition discrepancies.
(2) The proposed high-dimensional spatio-temporal feature topology graph utilizes spectral similarity-driven fusion to achieve multi-source information integration and spatial alignment, resolving the inadequate characterization of equipment spatio-temporal information and vibration transmission delays.
(3) The designed graph residual convolutional network integrating multi-source spatio-temporal features realizes high diagnostic performance and strong generalization capability under complex operating conditions by deeply mining spatio-temporal features and dynamically optimizing the topology structure.

The structure of this paper is organized as follows: Section 2 reviews related works on the application of topological graph structures in data feature extraction and spatio-temporal information mining from multi-source data. Section 3 provides a detailed description of the proposed methodology, including the graph construction and network architecture. Section 4 presents experimental validation of the proposed method’s effectiveness. Section 5 presents a sensitivity analysis of hyperparameters and benchmarks the proposed approach against representative methods. Section 6 concludes the paper.

## 2. Related Works

### 2.1. Structural Application of Topological Graph Structures in Data Feature Extraction

The topological structure refers to the connectivity relationships between nodes, characterizing the overall architectural features of a graph. This structure not only captures direct node-to-node connections but also reveals complex hidden relationships and patterns within the data. Topological structures are commonly implemented in domains such as bioinformatics, social networks, image processing, and natural language processing.

In feature extraction, feature topology employs graph models to represent the similarity, correlation, or interaction between features. By leveraging topological relationships among nodes, feature topology can effectively learn feature representations and better capture intrinsic data structures. As an example, Peng et al. [14] introduced a method for extracting image topological features and applied it to handwritten mathematical symbol recognition, achieving high classification accuracy. Liang et al. [15] optimized topological structures by extracting network features to improve communication network performance. Xuan et al. [16] enhanced graph learning through subgraph topology and dynamic graph topology while integrating pairwise feature contextual relationships for disease diagnosis and prediction.

This approach holds vital research significance and substantial implementation potential for diagnostic systems. By representing vibration signal features as graph nodes and establishing edges based on feature similarity metrics, the developed feature topology graph enables effective extraction of latent correlations in equipment failures under varying operational scenarios, which augments the model’s interpretative capability for fault patterns and consequently elevates the precision and resilience of diagnosis. Xu et al. [17] analyzed the topological structure of Petri nets to extract fault-related features, which were then integrated with machine learning algorithms for fault diagnosis. He et al. [18] employed a filter-bank topological structure to extract features associated with frictional impact faults from both displacement and acceleration signals, thereby enhancing feature representation and significantly improving diagnostic accuracy. Additionally, Jiang et al. [19] constructed an optimized feature topology graph from vibration data characteristics to refine inter-feature connectivity, subsequently integrating graph convolutional networks for imbalanced equipment fault diagnosis. These research findings demonstrate that feature topological information can more effectively characterize the dynamic evolution of data, particularly in complex operational scenarios, while establishing new research paradigms for fault diagnosis through topological feature representation.

### 2.2. Spatio-Temporal Information Mining from Multi-Source Data

In modern industrial systems, fault diagnosis is confronted with increasingly complex challenges. Multi-source data not only possesses intricate spatial relationships but also embed rich temporal dynamics, while variable operational conditions (such as changing speeds and loads) further compound data complexity. Therefore, spatio-temporal information from multi-source data and signals holds critical significance in fault diagnosis, as it synthesizes heterogeneous sensor or data-source information across spatial and temporal dimensions, thereby providing a more robust and comprehensive feature basis for fault modeling and identification.

Most existing feature-weighted fusion methods simply combine features from different data sources through operations like concatenation or weighted summation at intermediate model layers, lacking in-depth exploration of the underlying relationships between features. Therefore, developing fusion methods during the graph construction process is crucial for fully exploiting the latent information in multi-source data. For instance, Wen et al. [20] treated the positional relationships between different sensors as spatial correlations to construct the graph structure, while employing an attention module to model the significance of individual sensors. Xiang et al. [21] constructed two distinct topological structures based on distance metrics and similarity metrics, and then performed edge pruning according to structural similarity to achieve topological graph fusion. Miao et al. [22] developed a prior knowledge-based graph construction method that builds an association graph among sensors according to the mathematical model of wheeled robots, explicitly reflecting their physical interconnection relationships. Sun et al. [23] fused multi-source data into a spatio-temporal graph based on the tight connection mode of different sensors, as well as the different time steps and radius connections of the same sensor, which can capture broader spatial relationships and extract richer features. This approach enables comprehensive spatial relationship modeling and enhanced feature extraction capabilities. Current methods directly construct graph structures by fusing raw spatio-temporal data. While this approach preserves data integrity, it is prone to information redundancy due to inherent noise and interference in raw signals, resulting in insufficient feature extraction and ultimately compromising both spatio-temporal fusion effectiveness and the model’s diagnostic generalization capability under complex operating conditions.

To address these challenges, we propose a spatio-temporal collaborative perception-driven fusion framework for constructing spatio-temporal feature topology graphs. This approach effectively integrates multi-source spatio-temporal information to enable collaborative spatio-temporal understanding of heterogeneous data, significantly enhancing the granularity and comprehensiveness of feature mining.

## 3. Fault Diagnosis Methods for Rotating Equipment Under Complex Operating Conditions

### 3.1. Construction of Spatio-Temporal Feature Topology Graphs

#### 3.1.1. Construction of Single-Source Feature Topology Graphs

**Figure 1:**
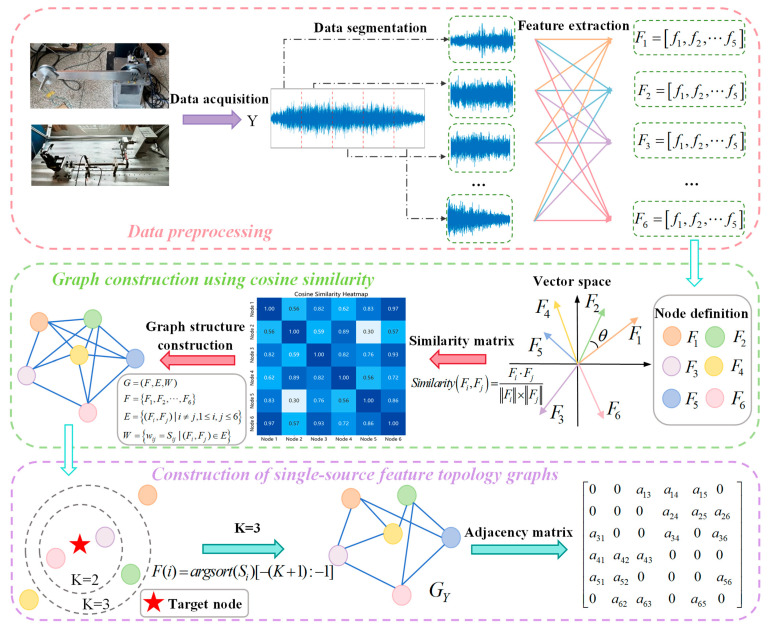
Construction of single-source feature topology graphs.

This paper introduces a feature correlation mining method based on topological graph structures, achieving more comprehensive feature representation through the construction of feature topology graphs. Specifically, we propose a similarity clustering-based approach that adaptively explores inter-feature relationships to construct feature topology graphs, as illustrated in Figure 1. The data utilized in Figure 1 originates from the normal-state dataset of the RV reducer in the triaxial varying-condition dataset described in Section 4, featuring six 5-dimensional feature nodes with the cluster number set to K = 3. Effective fault features form the foundation of fault diagnosis. Time- and frequency-domain features are widely recognized as capable of providing comprehensive diagnostic information. Accordingly, this study extracts 20 representative features from both domains, as detailed in Table 1, with 10 temporal descriptors and 10 spectral indicators. These encompass signal statistical characteristics, periodicity, energy distribution, and other critical aspects, effectively capturing the intrinsic patterns of fault signals.

Here, *n* corresponds to the length of the sampled time waveform, *n* = 768. $x_{i}$ represents the amplitude of the time-domain signal at index *i*, and $\bar{x}$ is the mean amplitude of the *n* data points. For frequency-domain analysis, *N* corresponds to the FFT output vector length, *N* = 384, $y_{k}$ and $p_{k}$ denote the raw spectral amplitude and its normalized counterpart for the k-th frequency component, respectively.

The proposed method represents single-source data as $x\in {\mathbb{R}}^{L\times 1}$, where *L* denotes the time-series length. First, the single-source data is segmented, and each segment is used to extract the aforementioned 20 features. These features serve as nodes in the graph, with each node’s feature vector composed of the same feature across different time steps, formulated as

(1) $$\left\{\begin{array}{c} F_{1}=\left[f_{1}\;f_{2}\;f_{3}\;f_{4}\cdots \cdots f_{n}\right]\\ F_{i}=\left[f_{1}\;f_{2}\;f_{3}\;f_{4}\cdots \cdots f_{n}\right]\\ \cdots \cdots \\ F_{20}=\left[f_{1}\;f_{2}\;f_{3}\;f_{4}\cdots \cdots f_{n}\right] \end{array}\right.$$

where *i* denotes the feature vector index, $i\in \left[1,20\right]$, and *n* represents the feature vector dimensionality, *n* = 50.

Subsequently, the cosine similarity is computed between single-source feature vectors using the following formula:

(2) $$Similarity(F_{i},F_{j})=\frac{F_{i}\cdot F_{j}}{\left\|F_{i}\right\|\times \left\|F_{j}\right\|}=\frac{\sum_{k=1}^{n}\left(F_{ik}\times F_{jk}\right)}{\sqrt{\sum_{k=1}^{n}{\left(F_{ik}\right)}^{2}}\times \sqrt{\sum_{k=1}^{n}{\left(F_{jk}\right)}^{2}}}$$

where $F_{i}$ and $F_{j}$ display the feature vectors at indices *i* and *j*, respectively, where *n* represents the feature vector dimensionality, *n* = 50, and *k* indicates the dimension index, $k\in \left[1,50\right]$. The similarity score range is [−1, 1], with values closer to 1 indicating higher node similarity.

This methodology adaptively determines the K most similar neighboring nodes for each node using the kNN algorithm. Empirical verification through iterative experiments established K = 7 as the optimal value, achieving an optimal trade-off between information richness and feature redundancy. This approach effectively enhances data representation capability while better accommodating the complex characteristics of sensor data in fault diagnosis tasks, ensuring stable model performance under varying operating conditions and providing a more informative basis for fault diagnosis. Through this process, we successfully constructed a topology graph that captures temporal feature relationships within single-source data, offering a novel perspective for analyzing feature sequence data. This graph serves as a critical foundation for subsequent spatio-temporal information fusion.

#### 3.1.2. Multi-Source Spatio-Temporal Information Fusion via Spectral Similarity

**Figure 2:**
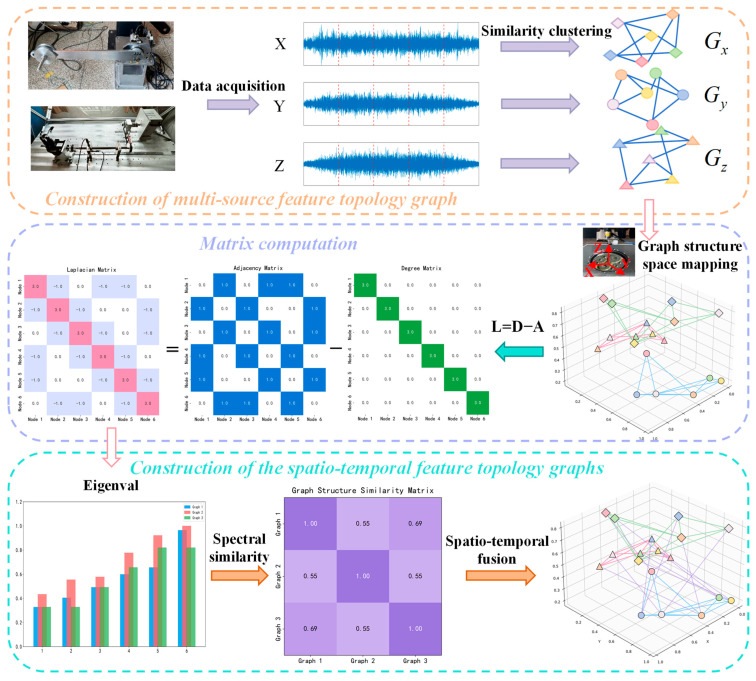
Construction of the spatio-temporal feature topology graphs.

After constructing single-source feature topology graphs, a critical challenge lies in effectively fusing multi-source spatio-temporal information to uncover underlying spatio-temporal relationships. Therefore, spectral similarity theory is introduced, treating the single-source feature topology graphs (constructed in Section 3.1.1) as subgraphs. By computing spectral similarity between these subgraphs, we achieve in-depth exploration of cross-source spatio-temporal correlations, thereby enabling effective fusion of multi-source spatio-temporal information, as illustrated in Figure 2.

For the subgraphs $G_{x}$, $G_{y}$, and $G_{z}$, we first compute their corresponding Laplacian matrices $L_{x}$, $L_{y}$, and $L_{z}$ using

(3) L=D−A

where *D* represents the node connectivity matrix with order 20, and *A* is the 20 × 20 adjacency matrix encoding node-edge connections. The Laplacian matrix captures the physical interpretation of signal smoothness on a graph. Smaller signal variations between neighboring nodes correspond to a smoother overall signal, indicating stronger spatial consistency or global coherence of fault features.

Next, we calculate the eigenvalue arrays $L_{x}$, $L_{y}$, and $L_{z}$ of the Laplacian matrices via

(4) $$eig_{i}=sort\left(real\left(eigvals\left(L_{i}\right)\right)\right)$$

where $i\in \left\{x,y,z\right\}$ denotes the subgraph index; $eigvals\left(\cdot \right)$ represents the eigenvalue decomposition of the Laplacian matrix; $real\left(\cdot \right)$ extracts the real part of eigenvalues; $sort\left(\cdot \right)$ arranges the real eigenvalues in ascending order. Lower-order eigenvalues characterize global, smooth modes of the graph, corresponding to holistic fault features, while higher-order eigenvalues capture local, rapidly varying components, reflecting subtle characteristics of localized faults.

Finally, the spectral similarity $sim_{xy}$, $sim_{xz}$, and $sim_{yz}$ between subgraphs is computed as

(5) $$similarity=\frac{1}{1+\sum_{m=1}^{k}\left|eig_{x}\left[m\right]-eig_{y}\left[m\right]\right|}$$

where *k* = 20 represents the graph order, and *m* denotes the eigenvalue index. $s\in \left[0,1\right]$, and the closer the value is to 1, the higher the similarity between subgraphs, indicating greater consistency in the distribution of fault patterns across the two graphs.

The connectivity threshold for feature topology graph interconnections is subsequently determined based on statistical principles. Given that similarity density distributions approximate a normal distribution, the threshold is set using the mean minus standard deviation method, denoted as θ=μ−σ. This threshold represents the lower bound of spectral similarity; only when the similarity between graph pairs exceeds θ are they considered structurally similar enough to establish an edge, weighted by their similarity value. Through this process, we successfully fuse multi-source spatio-temporal information to construct a spatio-temporal feature topology graph. This provides a robust foundation for subsequent model training and fault diagnosis tasks, enabling more accurate identification of fault patterns.

### 3.2. Spatio-Temporal Graph Residual Convolutional Neural Network

**Figure 3:**
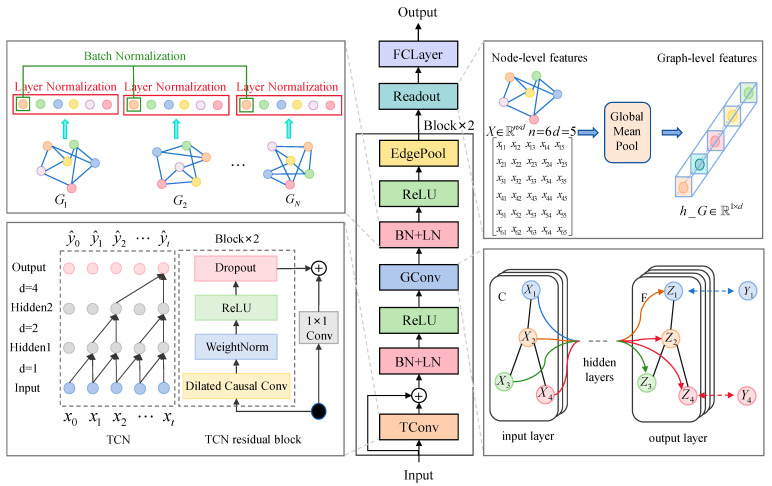
Spatio-temporal graph residual convolutional neural network.

To fully exploit spatio-temporal features within graph topologies and enhance fault diagnosis accuracy, we propose a graph residual convolutional neural network integrating multi-source spatio-temporal features, as illustrated in Figure 3. This network architecture primarily consists of temporal convolution blocks, graph convolution blocks, serial normalization layers (comprising batch normalization and layer normalization), readout layer, and fully connected layers. The spatio-temporal feature topological graph first passes through temporal convolution blocks to extract temporal features of each node, capturing complex dynamic variations along the time dimension. Subsequently, it undergoes graph convolution blocks to extract spatial features of nodes, obtaining integrated information through the aggregation of neighboring node features. After each convolution step, batch normalization is applied to stabilize batch-level statistical characteristics, followed by layer normalization to perform fine-grained scale calibration on features of individual nodes. This dual normalization strategy enables the model to simultaneously achieve cross-sample stability and cross-node adaptability, significantly enhancing its capacity to handle multi-scale features. Meanwhile, residual connections in temporal blocks prevent gradient vanishing. Subsequently, a readout layer is used to generate graph-level representations by pooling all node features, and, finally, two fully-connected layers are used for dimensionality reduction and classification. This integrated architecture enables hierarchical time–space feature learning while maintaining training stability through normalization techniques, ultimately achieving superior diagnostic performance through its end-to-end design, which processes raw spatio-temporal graphs directly into fault classification results.

### 3.3. Overall Framework

**Figure 4:**
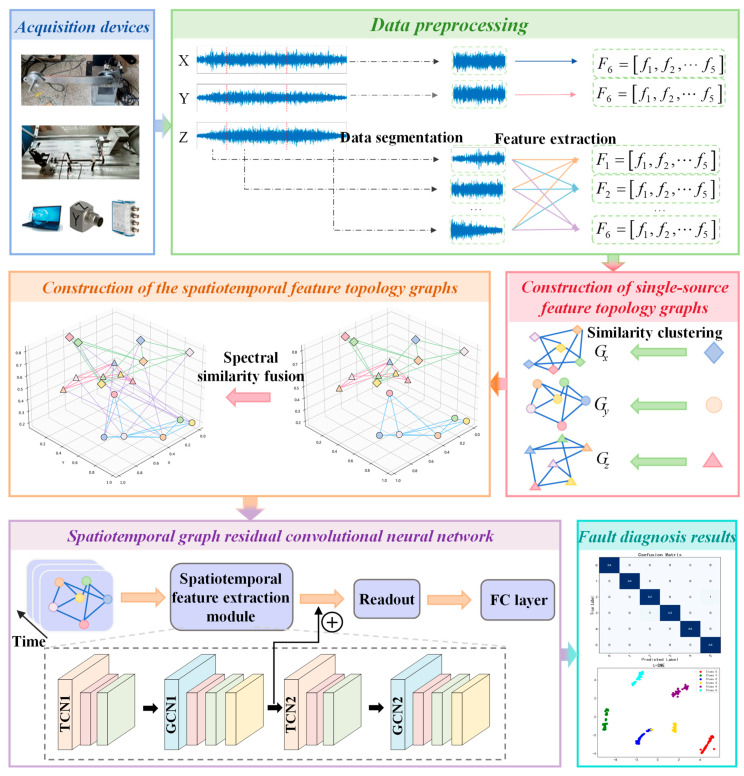
Overall process framework diagram.

This section outlines the key steps of the proposed multi-source spatio-temporal feature extraction method for complex operating conditions, as illustrated in Figure 4. The implementation procedure consists of five main stages:

Step 1. Data acquisition: multi-source data collection from RV reducers and SKF-6205 bearing test rigs using triaxial accelerometers, uniaxial sensors, NI-9234 DAQ modules, and cDAQ-9185 chassis.

Step 2. Data preprocessing: segmenting raw multi-source data and extracting representative features from each segment to form feature vectors.

Step 3. Multi-source feature topology graphs construction: representing features as graph nodes and building the topological structure through similarity clustering.

Step 4. Spatio-temporal feature topology graphs construction: fusing multi-source spatio-temporal information via spectral similarity to construct the integrated graph.

Step 5. Model training: the constructed spatio-temporal feature topological graphs, along with their corresponding fault category labels, are fed into the spatio-temporal graph residual convolutional neural network as inputs. Through supervised learning, the model is trained to output fault classification predictions.

## 4. Experimental Validation

### 4.1. Dataset Information

To rigorously validate the efficacy and general applicability of the proposed methodology, experiments were performed on two custom-built test platforms, generating two distinct datasets with different characteristics. The first dataset was acquired from triaxial sensors, exhibiting multidimensional, strongly coupled, and spatio-temporally unified properties that comprehensively reflect complex physical phenomena and motion states. The second dataset was collected from three uniaxial sensors, which differ in spatial transmission paths, producing relatively independent single-dimensional measurements that are commonly encountered in engineering applications.

Despite significant differences in data structure, feature distribution, and application scenarios between these two datasets, they both effectively capture equipment operational states under varying working conditions and contain critical information for fault diagnosis. The proposed method demonstrates robust performance across both datasets, showing consistent effectiveness in processing complex multidimensional data, as well as single-dimensional measurements. These results substantiate the method’s broad applicability and strong adaptability in handling diverse sensor data processing requirements.

#### 4.1.1. Variable Condition Triaxial Dataset

**Figure 5:**
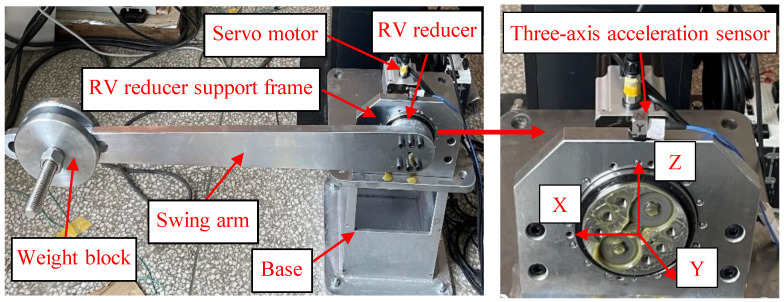
RV reducer test bench.

**Figure 6:**
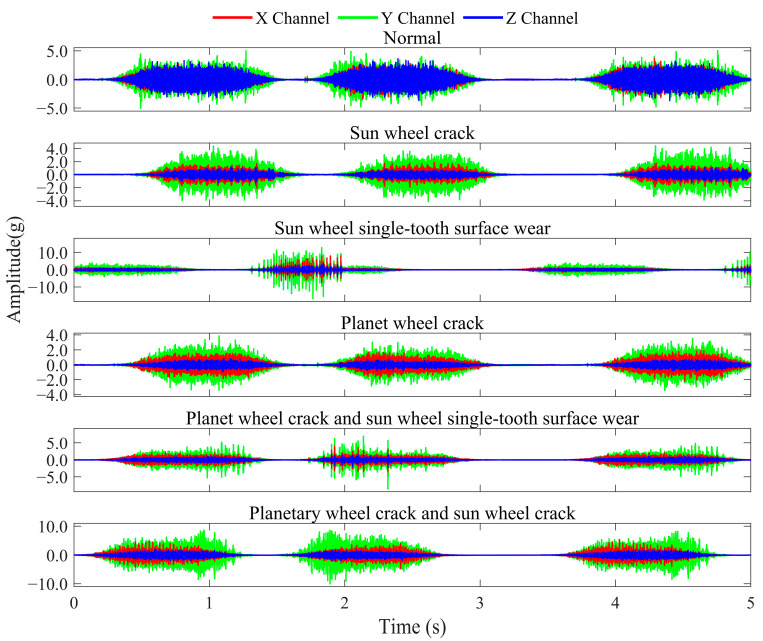
RV reducer waveform plot.

The RV reducer fault test bench is illustrated in Figure 5, consisting of five key components: the reducer, servo motor, support base, swing arm, and related fixtures. The load is applied by adding weight blocks at the tip of the swing arm. During testing, the servo motor drives the RV reducer’s swing arm to generate spatial vibrations while maintaining a 90° reciprocating motion at a speed of 100°/s on the output shaft. Data acquisition was performed using an NI-9234 DAQ module (National Instruments, Austin, TX, USA) with a triaxial accelerometer mounted at a fixed position, collecting synchronized vibration data across x, y, and z axes at a sampling rate of 25.6 kHz with 12,288 k data points, ensuring spatio-temporal synchronization and consistency. The experiments simulate potential faults in an RV reducer under variable operating conditions. These variable conditions comprise two distinct load cases: a 0 kg load and an 8 kg load, with each load configuration tested independently as a separate operating condition. Five types of faults are simulated: sun gear crack, single-tooth wear of the sun gear, planetary gear crack, combined planetary gear crack and single-tooth wear of the sun gear, and combined planetary gear crack and sun gear crack. The corresponding waveform plots are shown in Figure 6. As observed, the RV reducer’s motion during a swing arm cycle progresses through three distinct phases: initial acceleration, followed by constant speed, and final deceleration. This entire process is characterized by continuous speed variation, representing a continuous variable operating condition motion.

To examine the influence of variable operating conditions on fault characteristics while improving the model’s capability to recognize fault patterns under complex conditions and improve its generalization ability, this experiment treats all operational data under varying conditions for each fault type as a unified category for analysis. Consequently, the experimental setup constitutes a six-class classification task, as detailed in Table 2.

#### 4.1.2. Multi-Position Synchronous Source Dataset

**Figure 7:**
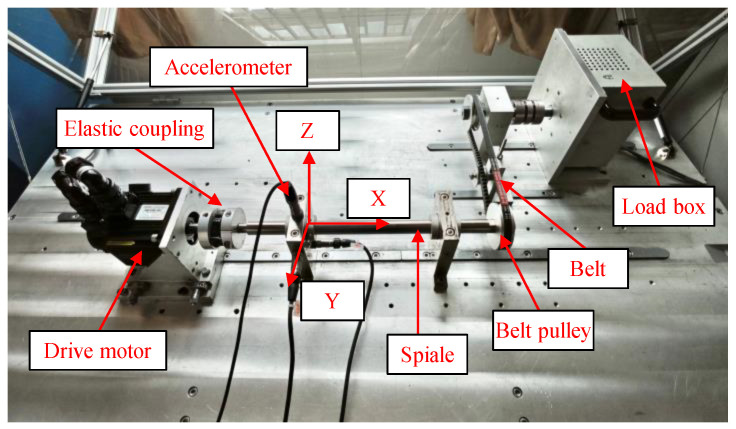
The SKF-6205 deep groove ball bearing test rig.

**Figure 8:**
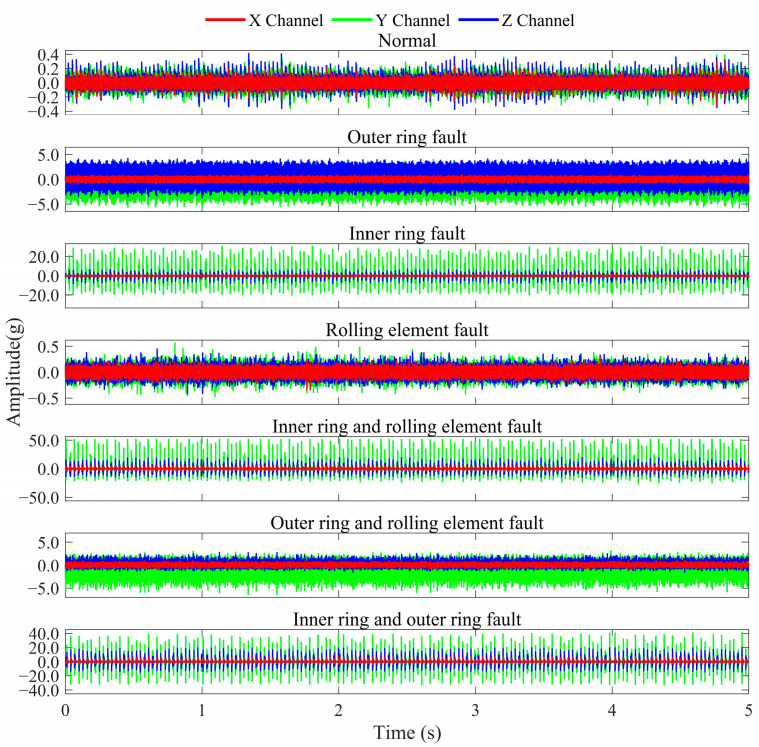
The SKF-6205 deep groove ball bearing waveform plot.

The SKF-6205 deep groove ball bearing test rig is shown in Figure 7, comprising a drive motor, flexible coupling, test bearing housing, rotating shaft, pulley, and magnetic powder brake loading unit. Speed and load are adjusted via the motor control software interface of the control cabinet, operating in constant-speed and constant-load mode. During experiments, the system operates at constant speed and load under the coordinated control of the drive motor and loading unit. Vibration data is collected using an NI-9234 DAQ module with three uniaxial accelerometers mounted at different positions, synchronously capturing x, y, and z-axis vibrations at a sampling rate of 25.6 kHz with 13,824 k data points to comprehensively characterize multi-directional vibration features. The experiments simulate potential bearing faults under multi-condition environments. The test matrix comprises variable speeds of 800 rpm, 2000 rpm, and 2400 rpm, combined with variable loads of 0 N, 3 N, and 6 N, resulting in nine unique operating conditions tested separately. Six fault types were examined: outer race faults, inner race faults, rolling element faults, compound faults involving inner race and rolling elements, compound faults involving outer race and rolling elements, and compound faults involving both inner and outer races. The corresponding waveform plots are shown in Figure 8, revealing that the bearing’s operational process exhibits stable multi-condition motion characteristics.

Similarly to the aforementioned experiment, this study consolidates all operational condition data for each fault type and analyzes them as a unified fault category. Consequently, the experiment constitutes a seven-class classification task, as specified in Table 3.

### 4.2. Experimental Details

In the experiments, spatio-temporal feature topological graphs were constructed as described in Section 3.1. The category label for each graph was predefined by the fault state of its corresponding vibration data. To address compound fault feature learning requirements while ensuring statistical significance, the dataset was partitioned into training and test sets at an 8:2 ratio. The Adam optimizer was employed for training with the cross-entropy loss function, and employed a training configuration with batch size = 32, 100 epochs, and learning rate = 0.005. The detailed model parameters are shown in Table 4. The experimental platform consisted of a Windows 11 operating system, an RTX 4060 GPU, Python 3.11.8 programming environment, and PyTorch 2.2.2 deep learning framework. It must be emphasized that the topological structure of the graphs is exclusively derived from signal features, while category labels serve solely as supervisory signals during model training and do not participate in the graph construction process.

To quantitatively validate the method’s effectiveness and cross-domain applicability, we selected accuracy and F1-score as evaluation metrics, while also employing confusion matrices and t-SNE visualization to validate classification performance. The evaluation metrics are mathematically formulated as follows:

Accuracy (Acc): The ratio of correctly predicted samples to total samples.

(6) $$Acc=\frac{TP+TN}{TP+FP+TN+FN}$$

where *TP* is correctly identified target cases, *TN* is correctly rejected non-target cases, *FP* is non-target cases erroneously flagged, *FN* is missed target cases.

F1-score: The harmonic mean of precision and recall, providing a balanced performance assessment.

(7) $$F1=2\times \frac{Precision\times Recall}{Precision+Recall}$$

With Precision and Recall calculated as

(8) $$Precision=\frac{TP}{TP+FP}$$

(9) $$Recall=\frac{TP}{TP+FN}$$

### 4.3. Analysis of Experimental Results

**Figure 9:**
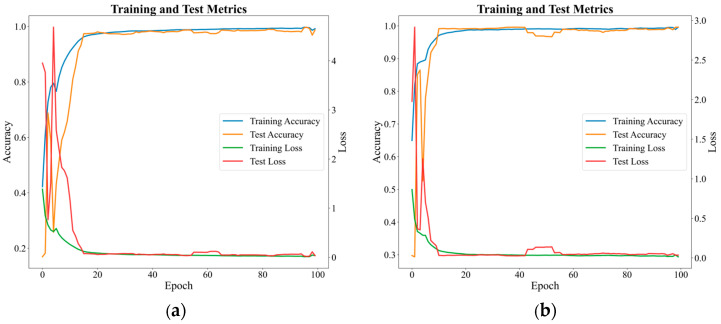
(**a**) The accuracy and loss curves of the proposed method with RV datasets. (**b**) The accuracy and loss curves of the proposed method with the SKF-6205 datasets.

The proposed method was evaluated on two distinct datasets. To demonstrate the stability and reliability of the method, 10 independent repeated trials were conducted on each dataset. The final metric was calculated by averaging the results from the last 30 training epochs per trial. As shown in Figure 9, the accuracy curves for both training and testing samples approach 1.0, while the loss values nearly converge to 0 with minimal fluctuations, indicating highly stable training dynamics. On the RV reducer dataset, the method achieved an average accuracy of 98.33% with a loss of 0.042; on the SKF-6205 bearing dataset, it attained an average accuracy of 97.39% with a loss of 0.0425. Furthermore, the method attained F1-scores of 0.9833 and 0.9738 on these datasets, demonstrating its excellent ability to balance precision and recall effectively. These findings collectively confirm that our method can accurately identify fault patterns while maintaining consistent performance across heterogeneous fault modalities, showcasing its robustness in handling diverse diagnostic scenarios.

**Figure 10:**
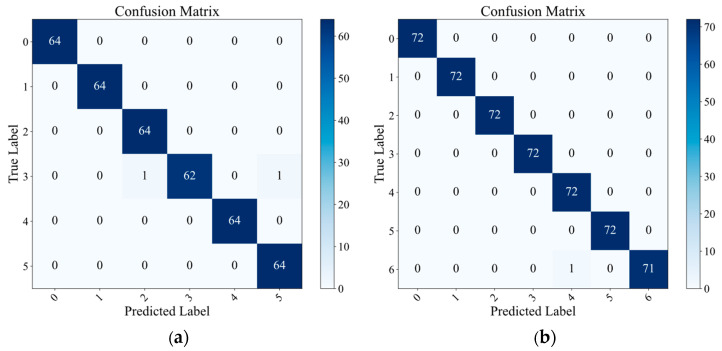
(**a**) The confusion matrices of the proposed method with RV datasets. (**b**) The confusion matrices of the proposed method with the SKF-6205 datasets.

**Figure 11:**
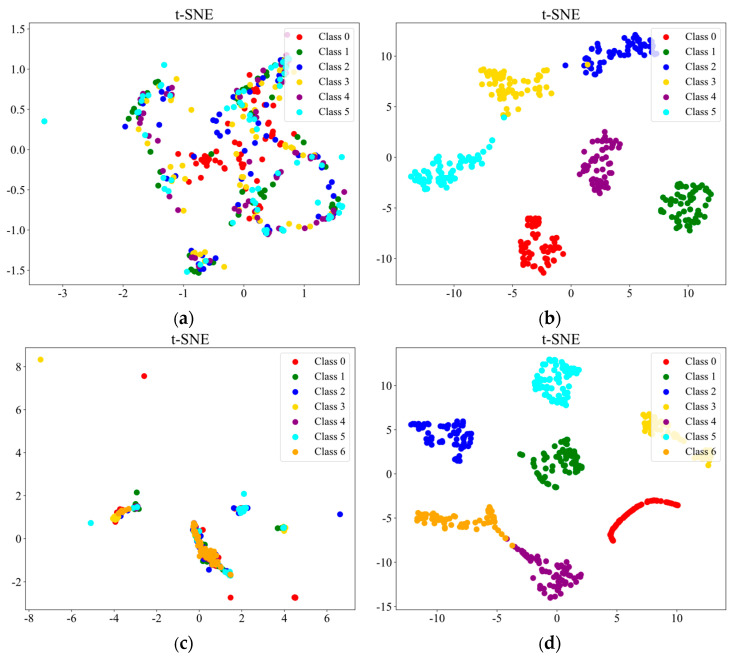
(**a**) Original feature distribution of the RV dataset. (**b**) Feature distribution of the RV dataset after applying the proposed method. (**c**) Original feature distribution of the SKF-6205 dataset. (**d**) Feature distribution of the SKF-6205 dataset after applying the proposed method.

To more intuitively demonstrate the classification efficacy of the proposed methodology in mechanical fault diagnosis and visualize the contrast between the original high-dimensional feature distribution and the processed feature distribution, we present the confusion matrices and t-SNE comparison plots for both datasets, as shown in Figure 10 and Figure 11. From the confusion matrices, it can be observed that the vast majority of the samples are correctly classified, with only a minimal number of misclassifications, demonstrating the strong generalization capability of the proposed method. The t-SNE comparison plots reveal that the original feature distributions of both datasets exhibit significant overlap and mixing, with unclear boundaries between different classes. However, after processing with the proposed method, the data points form distinct and well-separated clusters for each category. This further confirms that our method effectively extracts discriminative features from the graph structure, maintaining robust performance even in more complex datasets.

## 5. Further Discussion

### 5.1. Hyperparameter Analysis

#### 5.1.1. K Values Analysis

**Figure 12:**
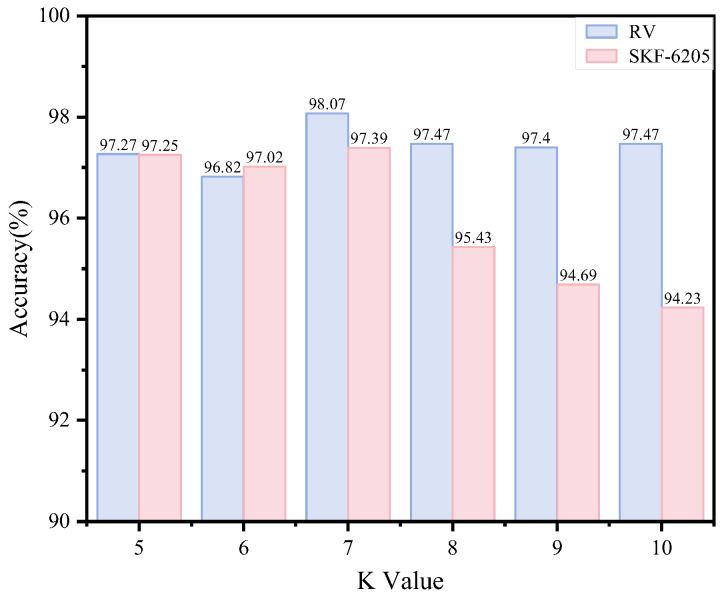
Model performance under different K values.

In our method, we employ cosine similarity combined with kNN to construct a graph structure incorporating spatio-temporal features. Different settings of the parameter K influence the conveyed feature information. To evaluate the stability of varying K values in the kNN algorithm within our framework, we systematically tested the classification performance across K values ranging from 5 to 10 for both datasets, as illustrated in Figure 12. The results demonstrate that as K increases, the model maintains consistently high performance on both datasets, with accuracy fluctuating within a narrow 2% margin, highlighting the method’s broad applicability. The optimal performance is achieved at K = 7. Beyond this value, a slight decline in accuracy occurs, attributable to redundant information introduced by excessive connections among neighboring feature nodes.

#### 5.1.2. Segment Length Analysis

**Figure 13:**
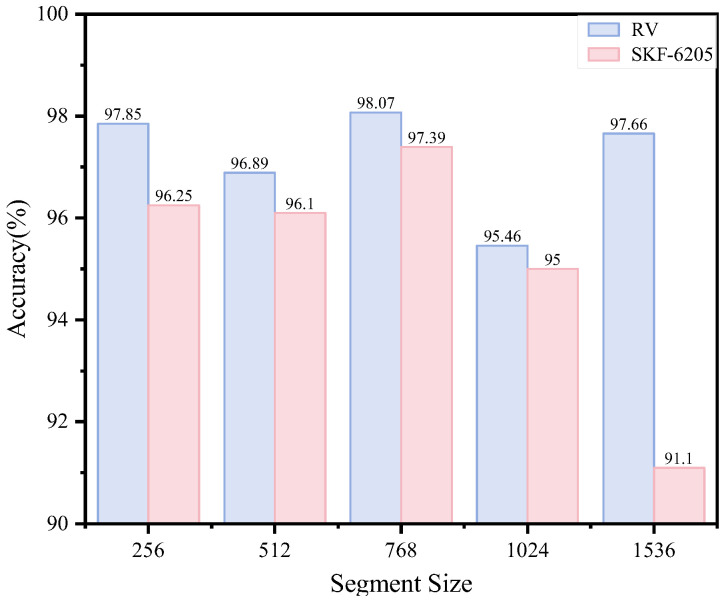
Model performance with segment length.

The segment length of raw data is a critical parameter that requires precise control during model construction—it must neither be too large nor too small. Excessively long segments increase input complexity and training time, while overly short segments fail to adequately capture key information from the original data. To evaluate the impact of segment length on our method, we tested varying segment lengths, as illustrated in Figure 13. The experimental results show that model accuracy improves with increasing segment length, peaking at 768 points, where optimal performance is achieved. However, beyond this threshold, accuracy declines as excessively long segments introduce additional noise and redundant information, leading to mild model overfitting.

#### 5.1.3. Feature Vector Dimensionality Analysis

**Figure 14:**
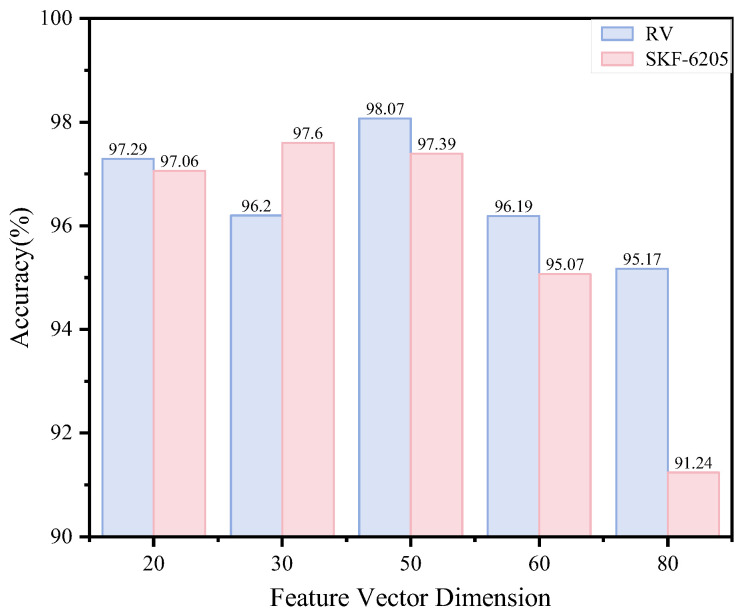
Model performance under feature vector dimensionality.

In our method, the feature vector dimension is set to 50. The dimensionality of feature vectors reflects both the complexity and information content of the data, while also determining the model’s computational complexity and generalization capability. To evaluate the impact of different feature dimensions on our method’s performance, we conducted experiments with varying dimensions and analyzed the corresponding accuracy rates, as shown in Figure 14. Experimental validation confirms that the model’s peak performance occurs at a feature dimensionality of 50, yielding the highest classification accuracy. In contrast, lower-dimensional features (e.g., 20 and 30 dimensions) show compromised performance due to insufficient flexibility in capturing the complex data distribution. When the feature dimension increases to 60 and 80, the model’s overall performance declines. This degradation occurs because higher-dimensional feature spaces increase model complexity and computational costs while introducing redundant information, ultimately making the model more prone to overfitting.

#### 5.1.4. Feature Type Analysis

**Figure 15:**
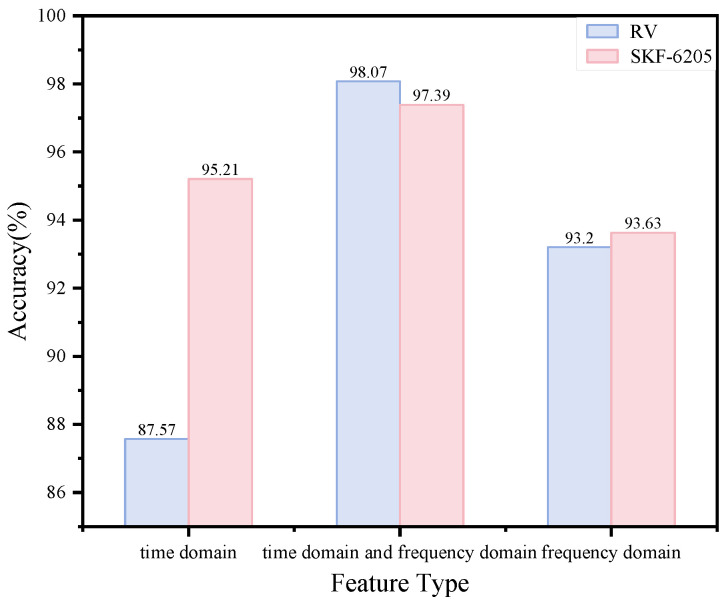
Model performance according to feature type.

In our methodology, we extract both time- and frequency-domain features that comprehensively characterize the original signal’s properties, including periodicity, energy distribution, sharpness, and other intrinsic characteristics. To evaluate the impact of using only a single type of feature, we conducted experiments on both datasets using either 10 temporal descriptors alone or 10 spectral indicators alone. The resulting classification performance is shown in Figure 15. The experimental results clearly demonstrate that models using either exclusively time-domain or frequency-domain features achieve significantly lower accuracy. This performance degradation occurs because single-type feature extraction fails to capture the complete information contained in the original signals. The resulting feature representations become informationally deficient, leading to inadequate model learning and poor generalization capability.

### 5.2. Ablation Study

To systematically evaluate the individual efficacy of each constituent module within the proposed method, we designed a series of ablation experiments and conducted evaluations on both datasets. We established four key module assessments:

- **Without feature extraction:** The single-source graph construction module employs a kNN-based approach using raw data directly.
- **Without cosine similarity and kNN graph construction:** The single-source graph construction module adopts a fully-connected feature-based approach.
- **Without spectral similarity fusion:** The multi-source graph structures remain unmerged, with three separate channels directly serving as model inputs.
- **Without temporal convolution block:** The model removes the temporal convolution block and utilizes a pure GCN architecture for fault identification.

The accuracy and F1-scores of the ablation experiments are presented in Table 5. The results demonstrate that the feature-based graph construction method outperforms the kNN-based approach using raw data, as it not only considers the original data characteristics but also overcomes the limited generalization capability of the kNN method. Compared to the fully-connected feature-based graph construction, the cosine similarity combined with the kNN approach better captures topological relationships between features while effectively filtering out redundant information. When compared to processing the three channels separately, the spectral similarity-based fusion of multi-source graphs enables deeper exploration of spatio-temporal features across different data sources. The proposed methodology effectively exploits the orthogonality of multidirectional vibration signatures, significantly enhancing the representation capability of spatio-temporal features. By incorporating the temporal convolution block, the model achieves a novel perspective for understanding and analyzing graph features across temporal and spatial domains, leading to superior classification performance.

### 5.3. Model Comparison

A comprehensive comparative analysis was performed between the proposed methodology and current state-of-the-art models to assess relative performance advantages, including 1D-CNN, Graph Attention Network (GAT) [24], Graph Isomorphism Network (GIN) [25], Higher-order Graph Convolutional Network (HoGCN) [26], Semi-supervised Graph Convolutional Network (SGCN) [27], Spectral Graph Convolutional Network (ChebyNet) [28], Spatial Graph Convolutional Network (GraphSage) [29], and Res-STGCN(X). A comparison of the model results is presented in Table 6. Compared with the classical non-graph model, 1D-CNN, the proposed method in this paper establishes a novel approach by constructing high-dimensional topological graph structures to model spatio-temporal relationships among multi-source data, achieving more accurate fault diagnosis. While GAT adaptively learns node features by assigning different weights to neighboring nodes, it struggles to effectively capture complex cross-source and cross-temporal dependencies. GIN emphasizes node feature aggregation capability but still faces challenges in handling variable operational conditions and complex temporal patterns for fault diagnosis tasks. SGCN demonstrates relatively weak overall performance, particularly on the SKF-6205 dataset, due to its rigid graph processing mechanism, which lacks fine-grained handling of local spatio-temporal features. Although HoGCN shows advantages in processing certain complex topological structures, it has limited capability in extracting periodic features from time-series fault data and learning node characteristics effectively. ChebyNet provides more flexible spectral filter approximations for multi-scale feature processing, yet fails to sufficiently learn the intrinsic structure of graph data in practical multi-source spatio-temporal scenarios. GraphSage performs well on both datasets, significantly outperforming other conventional graph neural networks, but its neighbor sampling and aggregation mechanism only preserves local spatio-temporal features while neglecting global temporal-spatial characteristics. In contrast to the single-channel Res-STGCN model, the multi-channel data construction in our method better represents spatial relationships between features, enabling the spatio-temporal graph model to effectively learn both temporal and spatial characteristics for accurate fault diagnosis.

## 6. Conclusions and Future Research

In summary, this paper proposes a multi-source spatio-temporal information fusion method for complex condition feature extraction, which effectively constructs spatio-temporal topological graph structures through similarity clustering and spectral similarity analysis. Additionally, we designed a graph residual convolutional neural network that integrates multi-source spatio-temporal features to enhance diagnostic performance by learning graph temporal characteristics. Through comprehensive experiments including ablation studies and model comparisons on two fundamentally different datasets, our proposed method demonstrates superior performance, outperforming non-graph models, single-axis models, and classical graph models in both accuracy and F1-score, particularly exhibiting strong potential in rotating machinery fault diagnosis scenarios where rich spatio-temporal coupling features exist and condition variations are detectable. This advantage primarily stems from its robust graph modeling capability.

However, our method relies on effective feature extraction and deep fusion of spatio-temporal information, and its applicability still requires further validation across a wider variety of equipment types and operating conditions. Therefore, in future research, we still need to focus on the following aspects:

1. Investigating more advanced graph feature mining methods to enhance the accuracy and robustness of topological graph structures in representing feature consistency.
2. Exploring more powerful multi-source information fusion techniques to improve the performance of high-dimensional topological graphs in processing spatio-temporal information.
3. Designing novel graph convolution units incorporating physical constraints and fault-sensitive gating tailored for variable operating condition diagnosis tasks, thereby enhancing the ability to capture spatio-temporal relationships and generalization across varying conditions.
4. Addressing the limited interpretability of GNNs by developing a causal-attention collaborative mechanism and interactive visualization, aiming to upgrade GNN-based diagnosis from post hoc attribution to real-time self-explanatory diagnosis.

## Figures and Tables

**Table 1:** Features proposed in this study.

Time-Domain Features		Frequency-Domain Features	
Crest Factor	$X_{CF}=\frac{\max(\left|x\right|)}{\sqrt{\frac{1}{n}}\sum_{i=1}^{n}\left|x_{i}\right|}$	Spectral Mean	$Y_{\mu }=\frac{1}{N}\sum_{k=1}^{N}\left|y_{k}\right|$
Impulse Factor	$X_{IF}=\frac{\max(\left|x\right|)}{\frac{1}{n}\sum_{i=1}^{n}\left|x_{i}\right|}$	Spectral Variance	$Y_{\sigma^{2}}=\frac{1}{N}{\sum_{k=1}^{N}\left(\left|y_{k}\right|-Y_{\mu }\right)}^{2}$
Time Kurtosis	$X_{K}=\frac{\frac{1}{n}\sum_{i=1}^{n}{\left(x_{i}-\bar{x}\right)}^{4}}{\sigma^{4}}$	Spectral Kurtosis	$Y_{K}=\frac{\frac{1}{N}{\sum_{k=1}^{N}\left(\left|y_{k}\right|-Y_{\mu }\right)}^{4}}{{\left(Y_{\sigma^{2}}\right)}^{2}}$
Form Factor	$X_{FF}=\frac{\sqrt{\frac{1}{n}\sum_{i=1}^{n}x_{i}^{2}}}{\frac{1}{n}\sum_{i=1}^{n}\left|x_{i}\right|}$	Spectral Skewness	$Y_{S}=\frac{1}{N}{\sum_{k=1}^{N}\left(\frac{\left|y_{k}\right|-Y_{\mu }}{Y_{\sigma }}\right)}^{3}$
Clearance Factor	$X_{M}=\frac{\max(\left|x\right|)}{{\left(\frac{1}{n}\sum_{i=1}^{n}\sqrt{\left|x_{i}\right|}\right)}^{2}}$	Spectral Centroid	$Y_{SC}=\frac{\sum_{k=1}^{N}k\cdot {\left|y_{k}\right|}^{2}}{\sum_{k=1}^{N}{\left|y_{k}\right|}^{2}}$
Root Mean Square	$X_{RMS}=\sqrt{\frac{1}{n}\sum_{i=1}^{n}x_{i}^{2}}$	Band Energy	$Y_{BE}=\sum_{k=start}^{end}{\left|y_{k}\right|}^{2}$
Peak-to-Peak Value	$X_{P-P}=\max(x)-\min(x)$	Spectral Entropy	$Y_{SE}=-\sum_{k=1}^{N}p_{k}\log_{2}(p_{k})$
Maximum Value	$X_{MAX}=\max(x)$	Dominant Frequency	$Y_{DF}=\arg\underset{k}{\max}\left|y_{k}\right|$
Standard Deviation	$X_{\sigma }=\sqrt{\frac{1}{n}\sum_{i=1}^{n}{\left(x_{i}-\bar{x}\right)}^{2}}$	Spectral Peak	$Y_{SP}=\underset{k}{\max}\left|y_{k}\right|$
Time Variance	$X_{\sigma^{2}}=\frac{1}{n}\sum_{i=1}^{n}{\left(x_{i}-\bar{x}\right)}^{2}$	Band Power	$Y_{BP}=\frac{1}{N}\sum_{k=start}^{end}{\left|y_{k}\right|}^{2}$

**Table 2:** RV reducer dataset specifications.

Operating Condition	Fault Type	Train Sample	Test Sample	Label
100°/s-0 kg 100°/s-8 kg	Normal	192	48	0
	Sun gear crack	192	48	1
	Sun gear single-tooth wear	192	48	2
	Planet gear crack	192	48	3
	Planet gear crack and sun gear single-tooth wear	192	48	4
	Planet gear crack and sun gear crack	192	48	5

**Table 3:** The SKF-6205 deep groove ball bearing dataset specifications.

Operating Condition	Fault Type	Train Sample	Test Sample	Label
800rpm-0N	Normal	288	72	0
800rpm-3N	Outer race fault	288	72	1
800rpm-6N	Inner race fault	288	72	2
2000rpm-0N	Rolling element fault	288	72	3
2000rpm-3N	Inner race fault and rolling element fault	288	72	4
2000rpm-6N	Outer race fault and rolling element fault	288	72	5
2400rpm-0N 2400rpm-3N 2400rpm-6N	Inner race fault and outer race fault	288	72	6

**Table 4:** Specific parameters of the spatio-temporal graph residual convolutional neural network.

Layer Name	Kernel Size	Output	Normalization	Activation
Input	-	1920 × 50	-	-
TCN1	50 × 50	1920 × 50	BN + LN	ReLU
GCN1	50 × 512	1920 × 512	BN + LN	ReLU
TCN2	512 × 512	1920 × 512	BN + LN	ReLU
GCN2	512 × 512	1920 × 512	BN + LN	ReLU
FC	512 × 256	1920 × 256	-	-
FC1	256×out_channel	1920×out_channel	-	-

**Table 5:** Ablation study.

Model	RV		SKF-6205	
	Acc	F1	Acc	F1
without feature extraction	0.9039	0.8877	0.9572	0.9533
without cosine similarity and kNN graph construction	0.922	0.9216	0.9543	0.9532
without spectral similarity fusion	0.9633	0.9619	0.9573	0.954
without a temporal convolution block	0.7745	0.7694	0.6572	0.6411
our method	0.9833	0.9833	0.9739	0.9738

**Table 6:** Model comparison.

Model	RV		SKF-6205	
	Acc	F1	Acc	F1
1D-CNN	0.7785	0.7333	0.4223	0.3632
GAT	0.8803	0.8777	0.7309	0.7223
GIN	0.8681	0.8681	0.7762	0.7754
SGCN	0.8296	0.8266	0.6643	0.6581
HoGCN	0.8213	0.8199	0.8475	0.8471
ChebyNet	0.809	0.7999	0.8215	0.7991
GraphSage	0.9368	0.934	0.9103	0.9065
Res-STGCN(X)	0.9592	0.9589	0.9203	0.919
Our model	0.9833	0.9833	0.9739	0.9738

## Data Availability

The data presented in this study are available upon request from the corresponding author. The data are not publicly available due to privacy concerns.
